# Publisher Correction: Real-Time Selective Sequencing with RUBRIC: Read Until with Basecall and Reference-Informed Criteria

**DOI:** 10.1038/s41598-019-53739-5

**Published:** 2019-11-20

**Authors:** Harrison S. Edwards, Raga Krishnakumar, Anupama Sinha, Sara W. Bird, Kamlesh D. Patel, Michael S. Bartsch

**Affiliations:** 1 Exploratory Systems Dept., Sandia National Laboratories, Livermore, CA USA; 2 Present Address: Institute of Biomaterials and Biomedical Engineering, University of Toronto, Toronto, Canada; 3 Systems Biology Dept., Sandia National Laboratories, Livermore, CA USA; 4 Biotechnology & Bioengineering Dept., Sandia National Laboratories, Livermore, CA USA; 5 Present Address: uBiome, San Francisco, CA USA; 6 Present Address: Purdue Partnerships Dept., Sandia National Laboratories, Albuquerque, NM USA

Correction to: *Scientific Reports* 10.1038/s41598-019-47857-3, published online 07 August 2019

The Article contains an error on the second line of Table 2 where “In-Threshold Reads/pore/min”

should read:

“In-Threshold Reads/min”

